# Onychomadesis-associated hand, foot, and mouth disease complicated by green nail syndrome in a pediatric patient: A case report and literature review

**DOI:** 10.1016/j.jdcr.2026.05.007

**Published:** 2026-05-12

**Authors:** Joshua Vance, Sahithi Talasila, Enea Gjoka, Wasim Nasir

**Affiliations:** a A.T. Still University Kirksville College of Osteopathic Medicine, Kirksville, Missouri; b Transitional Year Residency, HCA Florida Orange Park Hospital, Orange Park, Florida; c Dermatology & Cosmetic Center PLLC, Flint, Michigan

**Keywords:** Coxsackievirus, green nail syndrome, hand, foot, and mouth disease, nail disorders, onychomadesis, pediatric dermatology, Pseudomonas aeruginosa, skin of color

## Introduction

Hand, foot, and mouth disease (HFMD) is a common pediatric exanthem frequently caused by Coxsackie A16 and enterovirus 71, with Coxsackie A6 increasingly recognized in association with more severe and atypical presentations.^1^^,^^2^

Onychomadesis, defined as proximal detachment of the nail plate from the matrix due to transient nail matrix arrest, is reported as a delayed, self-limited sequela of HFMD in children, typically emerging 4-6 weeks after the acute illness.^1^^,^^2^

Green nail syndrome (GNS), or chloronychia, is an uncommon nail disorder caused by colonization with Pseudomonas aeruginosa, classically arising with chronic moisture exposure, distal onycholysis, or underlying nail disease such as onychomycosis.^3^ GNS typically manifests as a painless black-green to blue-green chromonychia resulting from Pseudomonas aeruginosa colonization within the subungual space. Some patients develop longitudinal bands or streaks that correspond to recurrent episodes of infection or trauma. In more advanced presentations, the nail plate may exhibit thickening, brittleness, or dystrophy with more extensive onycholysis.^3^

GNS condition occurs primarily in middle aged and older adults, most often between 40 and 60 years of age. Cases in individuals younger than 30 years are uncommon, and pediatric presentations are exceptionally rare, arising most often in the context of immunocompromise.^4^ Concomitant HFMD-associated onychomadesis and Pseudomonas GNS in a healthy child is exceedingly rare, and raises the question of if transient disruption of the nail matrix and nail plate, whether from viral inflammation, onychomadesis, or local trauma, can create a permissive microenvironment for opportunistic colonization by Pseudomonas aeruginosa, even in immunocompetent hosts.

We present the case of a previously healthy six-year-old female who developed onychomadesis following HFMD, subsequently complicated by GNS. To our knowledge, this represents one of the few cases of pediatric GNS, and the first reported case of GNS presenting following HFMD-associated onychomadesis.

## Case report

A previously healthy 6-year-old female presented with progressive proximal nail plate separation and new green discoloration of several toenails. One month prior to presentation, she had developed HFMD, characterized by oral ulcers and acral vesicles, which resolved without complication. Shortly after resolution of the HFMD, her mother noted painless loosening of several fingernails, followed by progressive proximal detachment of multiple fingernail and toenail plates. Over the subsequent weeks, the patient’s mother noted a green discoloration of the patient’s toenails. The patient denied any systemic symptoms, including fever or fatigue. She also denied any pain, pruritus, recent aquatic exposure, or recent travel.

**Fig 1:**
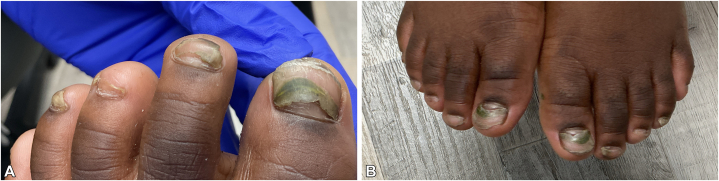
**A** and **B,** Chloronychia, otherwise known as green nail syndrome or Pseudomonas nail infection, presenting as well demarcated green to yellow-green chromonychia of the bilateral first-digit toenails with overlying scale and onychomadesis, accompanied by diffuse onychomadesis affecting multiple additional toenails bilaterally.

**Fig 2:**
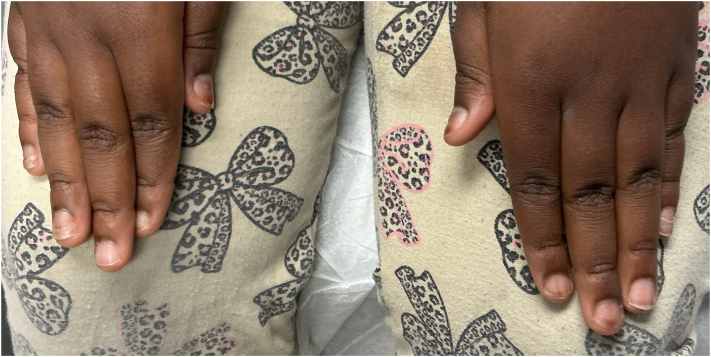
Onychomadesis presenting as proximally detached, shortened nail plates across multiple fingernails with emerging regrowth and melanonychia striata, without erythema, crusting, or secondary chromonychia.

Physical examination revealed that the patient’s right and left great toenails showed a well-circumscribed green to yellow-green chromonychia, consistent with GNS (Fig 1). Multiple fingernails and toenails bilaterally demonstrated onychomadesis, with complete separation of the proximal nail plate from the matrix (Fig 2).

The clinical findings were consistent with HFMD-associated onychomadesis, complicated by Pseudomonas aeruginosa nail infection. Given the characteristic green discoloration, a diagnosis of GNS was favored. No bacterial culture was obtained; the diagnosis of GNS was based on classic clinical findings and subsequent resolution with topical antipseudomonal therapy.

**Fig 3:**
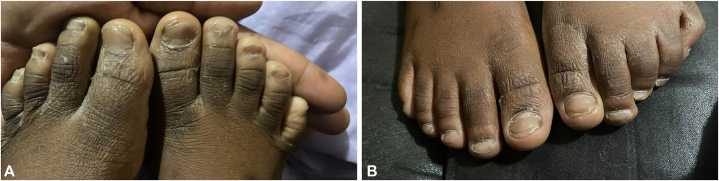
Clinical resolution of nail findings previously shown in Figs 1 and 2, characterized by normalization of nail coloration, continued proximal nail plate regrowth, and improvement in onychomadesis across multiple toenails, with residual periungual scale and mild nail dystrophy. Panels **A** and **B** show the same patient on the same day under different lighting conditions; both images are included to better demonstrate clinical findings.

The patient was prescribed gentamicin 0.1% ointment, and instructed to apply once daily to affected toenails. Follow-up demonstrated resolution of green nail discoloration with normal nail regrowth and no recurrence of onychomadesis or secondary infection (Fig 3).

## Discussion

A review of 4 published pediatric cases (Table I) demonstrates GNS arising in the setting of nail plate disruption from artificial nails, trauma, prolonged occlusion, or underlying immunocompromise, such as human immunodeficiency virus infection (Table I).^5^, ^6^, ^7^, ^8^ Together, these cases highlight that structural compromise of the nail unit may create a permissive environment for Pseudomonas colonization in children despite the overall rarity of GNS in this age group.

**Table I:** Demographics, clinical features, and outcomes of reported cases of pediatric green nail syndrome

Study name	Patient age and sex	Clinical presentation description	Risk factors or immunocompromise	Treatment
Nowakowska 2025^5^	**16; Female**	Green-brown discoloration localized to central nailbeds of all fingernails with mild yellow-green fluorescence under Wood's lamp; left eye corneal ulcer with severe thinning, central ring-shaped suppuration, and hypopyon	Prolonged home-applied artificial (press-on) nails (4 wk); contact lens wear	Oral ciprofloxacin (14 d) and 10 min bleach soaks twice daily
Corsello 2014^6^	**8; Male**	Green discoloration of the right toenails with exudative/erythematous skin lesions on the lower surface at pressure sites	HIV with CD4 count of 576 and HIV-RNA >100,000; chronic moisture exposure from unventilated athletic shoes	Topical neomycin/polymyxin B galenic unguentum applied twice daily
Urbonas 2025^7^	**8; Male**	Green discoloration of single fingernail with detachment	Nail trauma (finger slammed in car door); prolonged water exposure (daily swimming)	Topical mupirocin and regular drying of nailbed
Perz 2024^8^	**14; Female**	Asymptomatic green nail discoloration on several bilateral nail plates	Prolonged acrylic nail use (1 mo)	Topical gentamicin 0.3% under distal nail plates daily and vinegar soaks

*HIV*, Human immunodeficiency virus; *RNA*, ribonucleic acid.

In our patient, the juxtaposition of a pediatric viral exanthem with an infection typically seen in adults suggests that onychomadesis resulting from HFMD may contribute to conditions favorable for the development of GNS. Pathophysiologically, onychomadesis may predispose to GNS by creating a large proximal gap between the detached nail plate and the newly emerging nail plate that can trap water, soap, and debris, maintaining the warm, moist microenvironment required for Pseudomonas aeruginosa proliferation and biofilm formation.^9^ This biofilm results in the accumulation of pyocyanin/pyoverdin pigments on the undersurface of the nail plate which manifests as chloronychia. Soft tissue invasion is rare; however, in uncommon cases, Pseudomonas may spread into the nail bed epithelium, leading to protease-mediated tissue degradation and associated pain.^9^

Despite the plausibility of this mechanism, pediatric GNS remains rare. This suggests that transient arrest of matrix activity alone may be insufficient and that additional host or environmental factors likely influence colonization risk. Onychomycosis, a frequently cited predisposing condition for GNS, affects approximately 1% of children compared with 20% of adults older than 60.^10^ The relative infrequency of these susceptibility factors in children may contribute to the rarity of pediatric GNS, and highlights the unusual presentation in our otherwise healthy 6-year-old patient.

Although formal treatment guidelines for GNS have not been established, prior cases have shown successful clearance with both topical ointment and solution formulations of gentamicin. In 1 case series, topical 0.3% gentamicin solution applied nightly for 3 months was the most commonly used and effective treatment, while in our patient, 0.1% gentamicin ointment led to complete resolution.^9^ Other reported treatment options include antiseptic soaks and a range of topical antibiotics, while systemic antipseudomonal agents (aminoglycosides, fluoroquinolones, cephalosporins, carbapenems, and certain penicillins) are generally reserved for refractory or complicated cases with treatment guided by culture and sensitivity results.^9^

Further research is needed to define the age-related or immunologic factors and patterns of nail matrix injury, including HFMD and associated onychomadesis, that may predispose patients to the development of GNS. To our knowledge, this case represents one of few reported cases of GNS in an immunocompetent pediatric host, and the first reported instance of GNS following HFMD-associated onychomadesis. In the setting of HFMD, onychomadesis, or other nail matrix insults, clinicians should maintain a high index of suspicion for GNS in children and educate patients with onychomadesis on appropriate nail care and hygiene.

## Conflicts of interest

None disclosed.
